# The complete chloroplast genome sequence of *Helwingia himalaica* (Helwingiaceae, Aquifoliales) and a chloroplast phylogenomic analysis of the Campanulidae

**DOI:** 10.7717/peerj.2734

**Published:** 2016-11-29

**Authors:** Xin Yao, Ying-Ying Liu, Yun-Hong Tan, Yu Song, Richard T. Corlett

**Affiliations:** 1 Center for Integrative Conservation, Xishuangbanna Tropical Botanical Garden, Chinese Academy of Sciences, Xishuangbanna, Yunnan, China; 2 University of Chinese Academy of Sciences, Beijing, Beijing, China; 3 Key Laboratory of Dai and Southern medicine of Xishuangbanna Dai Autonomous Prefecture, Yunnan Branch Institute of Medicinal Plant, Chinese Academy of Medical Sciences, Jinghong, Yunnan, China

**Keywords:** Asterids, Campanulidae, Phylogeny, Plastomes, Yunnan

## Abstract

Complete chloroplast genome sequences have been very useful for understanding phylogenetic relationships in angiosperms at the family level and above, but there are currently large gaps in coverage. We report the chloroplast genome for *Helwingia himalaica*, the first in the distinctive family Helwingiaceae and only the second genus to be sequenced in the order Aquifoliales. We then combine this with 36 published sequences in the large (c. 35,000 species) subclass Campanulidae in order to investigate relationships at the order and family levels. The *Helwingia* genome consists of 158,362 bp containing a pair of inverted repeat (IR) regions of 25,996 bp separated by a large single-copy (LSC) region and a small single-copy (SSC) region which are 87,810 and 18,560 bp, respectively. There are 142 known genes, including 94 protein-coding genes, eight ribosomal RNA genes, and 40 tRNA genes. The topology of the phylogenetic relationships between Apiales, Asterales, and Dipsacales differed between analyses based on complete genome sequences and on 36 shared protein-coding genes, showing that further studies of campanulid phylogeny are needed.

## Introduction

Complete chloroplast genome sequences (plastomes) have been very useful for understanding phylogenetic relationships in angiosperms at the family level and above, and have been used to resolve previously recalcitrant nodes (Barrett et al., 2016). However, there are currently large gaps in the coverage of orders and families. Within the ‘very large, very old, and very widespread’ subclass Campanulidae (Beaulieu, O’Meara & Donoghue, 2013; also known as ‘Asterids II’), complete chloroplast genome sequences are currently available only for 74 species (out of c. 35,000), in six families (out of 29) and four orders (out of seven). Within the campanulid order Aquifoliales, plastome sequences are currently known only from the large, monogeneric family Aquifoliaceae (Yao et al., 2016).

*Helwingia* is the only genus in the campanulid family Helwingiaceae. It includes four species distributed in eastern Asia, from the Himalayas to Japan: *Helwingia chinensis* Batalin, *H. himalaica* Hook. f. & Thomson ex C.B. Clarke, *Helwingia japonica* (Thunb.) F. Dietr., and *Helwingia omeiensis* (W.P. Fang) H. Hara & S. Kurosawa (The Plant List, 2013; Wu, Raven & Hong, 2005). On current evidence, Helwingiaceae is sister to the Neotropical monogeneric family Phyllonomaceae (The Angiosperm Phylogeny Group, 2016), with which it shares an inferior ovary, epiphyllous inflorescence, and epigynous disc nectary (Ao & Tobe, 2015). These two small, highly disjunct, families are in turn sister to the near-cosmopolitan, but also monogeneric, family Aquifoliaceae.

Chloroplasts originated from free-living cyanobacteria via endosymbiosis and contain their own genome, which is circular and 76–217 kb in length (Hinsinger & Strijk, 2015; Zhang & Gao, 2016). Because of its abundance in plant cells and ease of sequencing, chloroplast DNA (cpDNA) has been widely utilized in studies of plant taxonomy and evolution (Kress et al., 2005; Kress & Erickson, 2007; Newmaster, Fazekas & Ragupathy, 2006; Chase et al., 2007; Taberlet et al., 2007). The small size, single unit, haploid nature, and highly conserved genomic structure of cpDNA also make it useful for species identifications (Yang et al., 2013). Moreover, the many copies per cell mean that useable fragments of the chloroplast genome are more likely to survive in dried herbarium specimens than are nuclear sequences, making direct comparisons with the genome of the type specimen potentially possible (Xu et al., 2015).

The Helwingiaceae’s current position in the order Aquifoliales, subclass Campanulidae (The Angiosperm Phylogeny Group, 2016), came after previous placements in the Cornaceae (Cronquist, 1981; Cronquist, 1988) and Araliaceae (Hutchinson, 1964; Hutchinson, 1973), and was based on molecular phylogenetic studies using *rbcL* (Morgan & Soltis, 1993), 18S rDNA and *rbcL* (Soltis & Soltis, 1997), and *ndhF* (Olmstead et al., 2000). Sequencing the chloroplast genome will facilitate the development of additional chloroplast markers for identification and phylogenetic studies within the family, as well as providing a basis for future studies on the phylogenetics and biogeography of the order Aquifoliales. Beaulieu, Tank & Donoghue (2013) suggest that the initial divergence within this order took place in Australasia in the Cretaceous, with an early expansion into South America and Asia where *Phyllonoma* and *Helwingia*, respectively, persist today, while *Ilex* has spread more widely. In the absence of a fossil record for the two small families, a higher resolution phylogeny is needed to assess this hypothesis. The wider phylogenetic relationships among campanulid orders have been investigated in several studies, using chloroplast markers only (Beaulieu, Tank & Donoghue, 2013; Wikström et al., 2015) or combined with nuclear ribosomal genes (ITS, 18S or 26S) (Tank & Donoghue, 2010; Beaulieu, O’Meara & Donoghue, 2013; Magallón et al., 2015), but not yet with complete chloroplast genomes.

Here, we first explore the structure of the chloroplast genome in the Helwingiaceae using *H. himalaica*. We then investigate the phylogenetic relationships in the Campanulidae by using the complete genome sequences and the protein-coding genes shared between *H. himalaica* and other published genomes.

## Materials and Methods

*Helwingia himalaica* is distributed from Nepal through northern India to southwestern China. Plants materials used in this study were intact, fresh, young leaves collected in Bingzhongluo county of Yunnan province (28.015306°N, 98.607944°E). The specimen has been deposited in the herbarium of the Xishuangbanna Tropical Botanical Garden, Chinese Academy of Sciences (HITBC). Total genomic DNA was extracted from fresh leaves using a modified CTAB method (Doyle, 1987; Yang, Li & Li, 2014). Each amplification was performed in 25 μL of a reaction mixture containing 1×PrimeSTAR GXL buffer (10 mM Tris-HCl (pH 8.2), 1 mM MgCl2, 20 mM NaCl, 0.02 mM EDTA, 0.02 mM DTT; 0.02% Tween 20, 0.02% Nonidet P-40, and 10% glycerol); 1.6 mM of dNTPs, 0.5 μM of each primer; 1.25 U of Prime-STAR GXL DNA polymerase (TAKARA BIO INC., Dalian, China), and 30–100 ng of DNA template. The amplification was conducted using 94 °C for 1 min, 30 cycles of 98 °C for 10 s and 68 °C for 15 min, followed by a final extension step at 72 °C for 10 min. The purified Polymerase chain reaction (PCR) product was fragmented and used for constructing the short-insert (500 bp) libraries according to the manufacturer’s manual (Illumina). DNA of each sample was then indexed by tags and pooled together in one lane in an Illumina Hiseq 2000 to sequence (Yang, Li & Li, 2014).

Raw reads were filtered by quality control software NGSQCToolkit v2.3.3 (Patel & Jain, 2012) to obtain high quality Illumina data (cut-off value for percentage of read length = 80, cut-off value for PHRED quality score = 30) and vector- and adaptor-free reads. Filtered reads were assembled into contigs in CLC Genomics Workbench v.8 (http://www.clcbio.com) by the de novo method using a *k*-mer of 63 and a minimum contig length of 1 kb. Outputted contigs were aligned with the chloroplast genome of the asterid *Camellia yunnanensis* (GenBank accession number: KF156838), which was the most similar genome identified via BLAST (http://blast.ncbi.nlm.nih.gov/), and ordered according to the reference genome. Genes in the assembled chloroplast genome were predicted using Dual Organellar GenoMe Annotator (DOGMA) (Wyman, Jansen & Boore, 2004). The chloroplast genome was assembled using aligned contigs in Geneious v. 8.1.7 (http://www.geneious.com, Kearse et al., 2012). Junctions between large single-copy (LSC)/inverted repeats (IRs) and small single-copy (SSC)/inverted repeats (IRs) were validated by Sanger sequencing of PCR-based products (Table S1).

The assembled genome was annotated using the DOGMA database (Wyman, Jansen & Boore, 2004), then manually edited for start and stop codons. Genome maps were drawn in OGDraw v.1.2 (Lohse et al., 2013). The annotated chloroplast genome has been submitted to GenBank (accession number: KX434807). REPuter was used to detect and assess repeats, including forward match, reverse match, complement match, and palindromic match repeats (Kurtz et al., 2001). Phobos v3.3.12 was used to detect simple sequence repeats (SSRs) under default parameters (Mayer, Christoph, Phobos 3.3.11, 2006–2010; http://www.rub.de/spezzoo/cm/cm_phobos.htm). Mauve v. 2.4.0 was used for determining the chloroplast genome rearrangements among the campanulid families (Darling et al., 2004).

A matrix of chloroplast genome sequences, including *H. himalaica*, 36 other campanulid species, and *Coffea arabica* as an outgroup (EF044213 in GenBank) (Table 1), was aligned using MAFFT (Katoh & Standley, 2013) and manually edited where necessary. These 37 campanulid species represent all families and major clades within the Campanulidae that had complete chloroplast genome sequences in GenBank. Unambiguously aligned DNA sequences were used for phylogeny construction. Phylogenies were constructed by maximum likelihood (ML), Bayesian Inference analyses (BI), and maximum parsimony (MP).

**Table 1:** List of campanulid species (and the outgroup, *Coffea arabica*) and their accession numbers in GenBank included in the phylogenetic analyses of whole chloroplast genomes.

Species	Accession number in NCBI	Family	Order	Length (bp)	Coding gene	tRNA	rRNA	GC (%)	LSC (bp)	SSC (bp)	IRs (bp)
*Angelica acutiloba*	KT963036	Apiaceae	Apiales	147,074	85	35	8	37.5	93,367 (63.48)	17,573 (11.95)	36,134 (24.57)
*Anthriscus cerefolium*	GU456628	Apiaceae	Apiales	154,719	85	37	8	37.4	84,768 (54.79)	17,551 (11.34)	52,400 (33.87)
*Bupleurum falcatum*	KM207676	Apiaceae	Apiales	155,989	84	37	8	37.7	85,870 (55.05)	17,518 (11.23)	52,601 (33.72)
*Crithmum maritimum*	HM596072	Apiaceae	Apiales	158,355	88	37	8	37.6	85,230 (53.82)	27,993 (17.68)	55,986 (35.35)
*Daucus carota*	DQ898156	Apiaceae	Apiales	155,911	85	43	8	37.7	84,244 (54.03)	17,571 (11.27)	54,096 (34.70)
*Foeniculum vulgare*	KR011054	Apiaceae	Apiales	153,628	85	37	8	37.6	86,659 (56.41)	17,470 (11.37)	49,499 (32.22)
*Ligusticum tenuissimum*	KT963039	Apiaceae	Apiales	158,500	88	37	8	37.6	84,875 (53.55)	17,661 (11.14)	55,964 (35.31)
*Ostericum grosseserratum*	KT852844	Apiaceae	Apiales	147,282	83	36	8	37.5	93,185 (63.27)	17,663 (11.99)	36,434 (24.74)
*Petroselinum crispum*	HM596073	Apiaceae	Apiales	152,890	84	37	8	37.8	86,116 (56.33)	17,508 (11.45)	49,266 (32.22)
*Tiedemannia filiformis subsp. greenmannii*	HM596071	Apiaceae	Apiales	154,737	85	37	8	37.3	84,585 (54.66)	17,140 (11.08)	53,012 (34.26)
*Dendropanax dentiger*	KP271241	Araliaceae	Apiales	156,687	87	37	8	38.0	86,680 (55.32)	18,247 (11.65)	51,760 (33.03)
*Hydrocotyle verticillata*	HM596070	Araliaceae	Apiales	153,207	85	37	8	37.6	84,352 (55.06)	18,739 (12.23)	50,116 (32.71)
*Kalopanax septemlobus*	KC456167	Araliaceae	Apiales	156,413	87	37	8	37.9	86,467 (55.28)	18,118 (11.58)	51,828 (33.14)
*Panax ginseng*	AY582139	Araliaceae	Apiales	156,318	87	37	8	38.1	86,114 (55.09)	18,070 (11.56)	52,134 (33.35)
*Ilex delavayi*	KX426470	Aquifoliaceae	Aquifoliales	157,671	95	40	8	37.6	87,000 (55.18)	18,436 (11.69)	52,234 (33.13)
*Ilex latifolia*	KX426465	Aquifoliaceae	Aquifoliales	157,610	95	40	8	37.6	86,952 (55.17)	18,429 (11.69)	52,228 (33.14)
*Ilex* new species	KX426469	Aquifoliaceae	Aquifoliales	157,611	95	40	8	37.6	86,948 (55.17)	18,434 (11.70)	52,227 (33.14)
*Ilex polyneura*	KX426468	Aquifoliaceae	Aquifoliales	157,621	95	40	8	37.6	87,064 (55.24)	18,435 (11.70)	52,122 (33.07)
*Ilex pubescens*	KX426467	Aquifoliaceae	Aquifoliales	157,741	95	40	8	37.6	87,109 (55.22)	18,436 (11.69)	52,238 (33.12)
*Ilex szechwanensis*	KX426466	Aquifoliaceae	Aquifoliales	157,822	95	40	8	37.7	87,204 (55.25)	18,513 (11.73)	52,182 (33.06)
*Ilex wilsonii*	KX426471	Aquifoliaceae	Aquifoliales	157,918	95	40	8	37.6	87,266 (55.26)	18,432 (11.67)	52,222 (33.07)
*Helwingia himalaica*	KX434807	Helwingiaceae	Aquifoliales	158,362	94	40	8	37.7	87,810 (55.45)	18,560 (11.72)	51,991 (32.83)
*Artemisia frigida*	JX293720	Asteraceae	Asterales	151,076	87	37	8	37.5	82,740 (54.77)	18,392 (12.17)	49,944 (33.06)
*Aster spathulifolius*	KF279514	Asteraceae	Asterales	149,510	87	37	8	37.7	81,961 (54.82)	17,972 (12.02)	49,577 (33.16)
*Centaurea diffusa*	KJ690264	Asteraceae	Asterales	152,559	90	36	8	37.7	83,596 (54.80)	18,487 (12.12)	50,476 (33.09)
*Chrysanthemum indicum*	JN867592	Asteraceae	Asterales	151,129	85	35	8	37.4	82,885 (54.84)	18,376 (12.16)	49,868 (33.00)
*Cynara cornigera*	KP842707	Asteraceae	Asterales	152,550	87	37	8	37.7	83,580 (54.79)	18,660 (12.23)	50,310 (32.98)
*Lactuca sativa*	DQ383816	Asteraceae	Asterales	152,772	86	44	8	37.5	84,105 (55.05)	18,599 (12.17)	50,068 (32.77)
*Lasthenia burkei*	KM360047	Asteraceae	Asterales	150,944	67	25	7	37.4	82,193 (54.45)	18,271 (12.10)	50,480 (33.44)
*Parthenium argentatum*	GU120098	Asteraceae	Asterales	152,803	57	17	8	37.6	84,593 (55.36)	18,900 (12.37)	49,310 (32.27)
*Praxelis clematidea*	KF922320	Asteraceae	Asterales	151,410	84	32	8	37.2	85,311 (56.34)	18,559 (12.26)	47,540 (31.40)
*Adenophora remotiflora*	KP889213	Campanulaceae	Asterales	171,724	82	37	8	38.8	105,555 (61.47)	11,295 (6.58)	54,874 (31.95)
*Campanula takesimana*	KP006497	Campanulaceae	Asterales	169,551	83	36	8	38.8	102,320 (60.35)	7,747 (4.57)	59,484 (35.08)
*Hanabusaya asiatica*	KJ477692	Campanulaceae	Asterales	167,287	82	37	10	38.8	104,955 (62.74)	8,578 (5.13)	53,754 (32.13)
*Trachelium caeruleum*	EU090187	Campanulaceae	Asterales	162,321	83	44	10	38.3	100,110 (61.67)	7,661 (4.72)	54,550 (33.61)
*Kolkwitzia amabilis*	KT966716	Caprifoliaceae	Dipsacales	156,875	81	38	8	38.4	90,137 (57.46)	18,846 (12.01)	47,892 (30.53)
*Lonicera japonica*	KJ170923	Caprifoliaceae	Dipsacales	155,078	81	39	8	38.6	88,858 (57.30)	18,672 (12.04)	47,548 (30.66)
*Coffea arabica*	EF044213	Rubiaceae	Gentianales	155,189	85	45	8	37.4	85,164 (54.88)	18,207 (11.73)	51,818 (33.39)

**Note:**

Numbers in parentheses in the LSC, SSC and IRs columns are the percentage of the total length.

ML analyses were conducted in RAxML version 8.2.8 (Stamatakis, 2014), using the GTACAT approximation. Convergence of the bootstrap was tested in RAxML using a posteriori bootstrapping analysis. BI analysis was conducted using MrBayes version 3.2.6 (Ronquist et al., 2012) and the best substitution model (‘TVM+G’) tested by AIC in jModelTest version 2.1.10 (Darriba et al., 2012). Four independent Markov Chain Monte Carlo algorithms were calculated for 10,000,000 generations and sampled every 1,000 generations. Potential Scale Reduction Factor (PSRF) values were used to determine convergence in BI using MrBayes version 3.2.6. All PSRF values were 1, indicating that these analyses converged. The first 25% of calculated trees was discarded as burn-in and a consensus tree constructed using the remaining trees. MP analysis was conducted PAUP version 4.0a150 (http://people.sc.fsu.edu/~dswofford/paup_test/), using the heuristic searches with tree bisection-reconnection (TBR) branch swapping and the ‘Multrees’ option in effect. Bootstrap analysis was conducted with 1,000 replicates with TBR branch swapping.

In addition, 36 protein-coding genes (Table 1) shared across all the 37 campanulid species were selected to build the phylogeny. ML analyses were conducted in RAxML version 8.2.8 (Stamatakis, 2014), using the GTACAT approximation. Convergence of the bootstrap was tested in RAxML using a posteriori bootstrapping analysis. BI analysis was conducted using MrBayes version 3.2.6 (Ronquist et al., 2012) and the best substitution model (‘GTR+I+G’) tested by AIC in jModelTest version 2.1.10 (Darriba et al., 2012). Methods for phylogeny construction using the 36 protein-coding genes follow the description above. PSRF values were used to determine convergence in BI using MrBayes version 3.2.6. All PSRF values were 1, indicating that these analyses converged. The first 25% of calculated trees was discarded as burn-in and a consensus tree constructed using the remaining trees. MP analysis was conducted in PAUP version 4.0a150 (http://people.sc.fsu.edu/~dswofford/paup_test/), using the heuristic searches with TBR branch swapping and the ‘Multrees’ option in effect. Bootstrap analysis was conducted with 1,000 replicates with TBR branch swapping.

## Results

### Genome features

**Figure 1:**
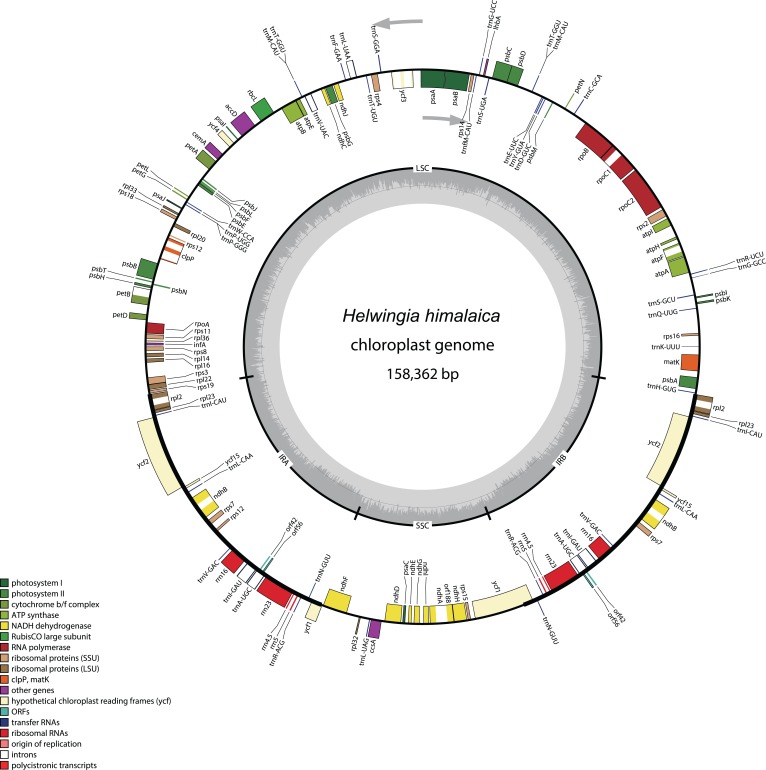
Circular gene map of the *Helwingia himalaica* chloroplast genome. Genes placed outside of the outer circle are transcribed in clockwise direction whereas genes inside are transcribed in counterclockwise direction. Different colours refer to genes from different functional groups. The area in darker gray in the inner circle indicates GC content while the lighter gray indicates AT content.

The total length of the chloroplast genome is 158,362 bp. Its quadripartite structure includes an LSC with 87,810 bp and SSC with 18,560 bp, separated by a pair of IR regions with lengths of 25,996 bp (Fig. 1). The GC content is 37.7% (see Table 1 in Yao et al., 2016). A total of 102 unique genes were detected in the chloroplast genome, of which 20 were duplicated in IR regions. Totally, 94 protein-coding genes (76 unique) encode proteins acting in processes related to photosynthesis, the genetic system, and some currently unknown functions (e.g. *ycf*). In addition, 40 genes (26 unique) encode for tRNAs and eight genes for rRNAs (Table 2). All eight rRNA genes are in IR regions. One *ycf1* gene is a functional pseudogene as it is on the border between the SSC and IRa region. Gene *rps19* is outside the IRb region at the LSC-IRb junction and *rpl2* is fully included in the IRa region. Five genes (*atpF*, *rpoC1*, *rpl2*, *ndhB* and *ndhA*) have one intron and two genes have two introns (*ycf3*, *clpP* and *rps12*).

**Table 2:** List of genes in the chloroplast genome of *Helwingia himalaica*.

Category	Groups of gene	Name of genes
Protein synthesis and DNA-replication	Transfer RNAs	*trnC-GCA, trnD-GUC, trnE-UUC, trnF-GAA, trnfM-CAU, trnG-GCC, trnG-UCC, trnH-GUG, trnK-UUU, trnL-UAA, trnM-CAU, trnQ-UUG, trnP-GGG, trnP-UGG, trnR-UCU, trnS-GCU, trnS-GGA, trnS-UGA, trnT-GGU, trnT-UGU, trnV-UAC, trnW-CCA, trnY-GUA, trnA-UGC(×2), trnI-CAU(×2), trnI-GAU(×2), trnL-CAA(×2), trnL-UAG, trnN-GUU(×2), trnR-ACG(×2), trnV-GAC(×2)*
	Ribosomal RNAs	*rrn16(×2), rrn23(×2), rrn4.5(×2), rrn5(×2)*
	Ribosomal protein small subunit	*rps16, rps2, rps14, rps4, rps18, rps12(×2), rps11, rps8, rps3, rps19, rps7(×2), rps15*
	Ribosomal protein large subunit	*rpl33, rpl20, rpl36, rpl14, rpl16, rpl22, rpl2(×2), rpl23(×2), rpl32*
	Subunits of RNA polymerase	*rpoA, rpoB, rpoC1, rpoC2*
Photosynthesis	photosystem I	*psaA, psaB, psaC, psaI, psaJ*
	Photosystem II	*psbA, psbB, psbC, psbD, psbE, psbF, psbG, psbH, psbI, psbJ, psbK, psbL, psbM, psbN, psbT, lhbA*
	Cythochrome b/f complex	*petA, petB, petD, petG, petL, petN*
	ATP synthase	*atpA, atpB, atpE, atpF, atpH, atpI*
	NADH-dehydrogenase	*ndhA, ndhB(×2), ndhC, ndhD, ndhE, ndhF, ndhG, ndhH, ndhI, ndhJ, ndhK*
	Large subunit rubisco	*rbcL*
Miscellaneous group	Translation initiation factor	*infA*
	Acetyl-CoA carboxylase	*accD*
	Cytochrome c biogenesis	*ccsA*
	Maturase	*matK*
	ATP-dependent protease	*clpP*
	Inner membrane protein	*cemA*
Pseudogene unknown function	Conserved hypothetical chloroplast ORF	*ycf3, ycf4, ycf2(×2), ycf15(×2), orf42(×2), orf56(×2), ycf1(×2), orf188*

### Repeated sequences and SSR

Thirty repeated sequences were detected, with lengths ranging from 18 to 43 bp and sequence identity more than 90% (Table 3). Among them, 19 repeated sequences were dispersed in intergenic regions, 10 in genes, and one in introns. There were 16 forward repeats, nine palindromic repeats, three reverse repeats, and two complement repeats, and 21, 2 and 7 repeats were detected in the LSC, SSC and IRs, respectively. A total of 813 SSRs were found, including 289 mononucleotides, 35 dinucleotides and 70 trinucleotides (Fig. S1). In mononucleotide SSRs, thymine and adenine made up 92% (266). In dinucleotide SSRs, we found repeated units consisting of TA/AT and GA/AG, but no GC/CG and TC/CT repeats.

**Table 3:** List of repeated sequences in the chloroplast genome of *Helwingia himalaica*.

Repeat length (bp)	Repeat A start site	Repeat A location*	Repeat A region	Repeat B start site	Repeat B location	Repeat B region	Repeat type**
43	0	*rpl2(trnH-GUG)*	IRa	87797	*rps19(rpl2)*	IRb	P
30	9030	*trnS-GCU*	LSC	47728	*trnS-GGA*	LSC	P
27	45989	*ycf3 intron2*	LSC	124368	*ndfA intron*	SSC	F
26	43	*rpl2(trnH-GUG)*	LSC	87772	*rps19(rpl2)*	IRb	P
26	10811	*trnG-GCC(trnR-UCU)*	LSC	10840	*trnG-GCC(trnR-UCU)*	LSC	P
26	33886	*trnT-GGU(psbD)*	LSC	33912	*trnM-CAU(psbD)*	LSC	F
26	91380	*ycf2*	IRb	154796	*ycf2*	IRa	F
23	61775	*accD*	LSC	61786	*accD*	LSC	F
21	9036	*trnS-GCU*	LSC	37766	*trnS-UGA*	LSC	F
21	37766	*trnS-UGA*	LSC	47731	*trnS-GGA*	LSC	P
21	38950	*trnM-CAU*	LSC	69860	*trnP-UGG*	LSC	F
20	38564	*lhbA(trnG-UCC)*	LSC	38581	*lhbA(trnG-UCC)*	LSC	F
20	49313	*trnT-UGU(trnL-UAA)*	LSC	49333	*trnT-UGU(trnL-UAA)*	LSC	F
19	385	*trnH-GUG(psbA)*	LSC	412	*trnH-GUG(psbA)*	LSC	P
19	6791	*rps16(trnQ-UUG)*	LSC	6817	*rps16(trnQ-UUG)*	LSC	F
19	8756	*psbI*	LSC	38919	*trnG-UCC(trnfM-CAU)*	LSC	P
19	10620	*trnG-GCC*	LSC	38738	*trnG-UCC*	LSC	F
19	15636	*atpH(atpI)*	LSC	15653	*atpH(atpI)*	LSC	F
19	34034	*trnT-GGU(psbD)*	LSC	111456	*orf56(trnR-ACG)*	IRb	R
19	34034	*trnT-GGU(psbD)*	LSC	134727	*trnR-ACG(trnA-UGC)*	IRa	C
19	53790	*ndhC(trnV-UAC)*	LSC	81476	*rpoA*	LSC	P
19	59571	*rbcL*	LSC	59590	*rbcL(accD)*	LSC	F
18	4719	*trnK-UUU*	LSC	66845	*petA(psbJ)*	LSC	R
18	5785	*rps16(trnQ-UUG)*	LSC	34036	*trnT-GGU(psbD)*	LSC	R
18	6349	*rps16(trnQ-UUG)*	LSC	90529	*ycf2*	IRb	F
18	6349	*rps16(trnQ-UUG)*	LSC	155655	*ycf2*	IRa	P
18	9101	*trnS-GCU*	LSC	37836	*trnS-UGA*	LSC	F
18	40424	*psaB*	LSC	42639	*psaA*	LSC	F
18	40973	*psaB*	LSC	43197	*psaA*	LSC	F
18	57793	*atpB(rbcL)*	LSC	121171	*ndhD(psaC)*	SSC	C

**Notes:**

* *rpl2*(*trnH-GUG*) means spacer between *rpl2* and *trnH-GUG*, *etc.*

** P means palindromic match, F means forward (direct) match, R means reverse match, and C means complement match.

### Genome rearrangement in the Campanulidae

**Figure 2:**
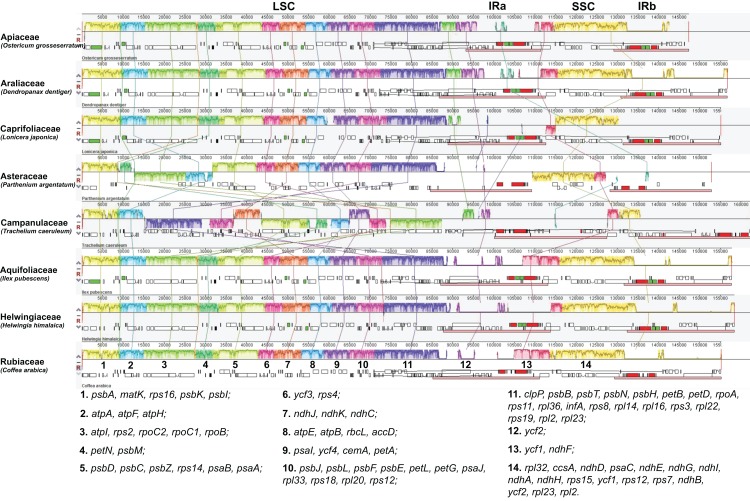
Gene arrangement map of chloroplast genome alignment of seven representative species from seven campanulid families and *Coffea arabica* (as a reference) determined by Mauve software (Darling et al., 2004). The polyline in the blocks indicates sequence similarity among these eight species. Line linking gene blocks among the eight species with same colour indicates ortholog. Gene blocks above are transcribed clockwise and those below are transcribed counterclockwise. The coding genes in the 14 main gene blocks are listed under the figure.

Genome alignment among seven species from the seven campanulid families with known chloroplast genomes revealed massive gene rearrangement, especially in the LSC (Fig. 2). Moreover, all four Campanulaceae species had longer genomes and LSCs, and shorter SSCs, compared with other campanulid species (Table 1). The IR in the chloroplast genome of some Apiaceae (*Angelica acutiloba*, *Foeniculum vulgare*, *Ostericum grosseserratum* and *Petroselinum crispum*) was contractive (Table 1; Fig. 2). Even though the lowest number of coding genes in any campanulid species was 57 (*Parthenium argentatum*), only 36 coding genes were shared across all the campanulid families (Table 4), Kumar et al. (2009) which indicates many gene losses or gains had occurred. The number of tRNA ranged from 17 (*P. argentatum*) to 44 (*Lactuca sativa* and *Trachelium caeruleum)*, while the number of rRNA was usually eight (Table 1).

**Table 4:** The 36 protein-coding genes shared by the 37 campanulid species and used for construction of the protein-coding gene phylogeny.

Gene	Length (bp)	Gene	Length (bp)	Gene	Length (bp)
*atpA*	1,539	*psaB*	2,205	*psbT*	144
*atpH*	246	*psaC*	246	*rbcL*	1,458
*atpI*	744	*psaI*	113	*rpl14*	417
*cemA*	708	*psaJ*	135	*rpl20*	415
*ndhC*	363	*psbA*	1,062	*rpl32*	207
*ndhD*	1,516	*psbD*	1,062	*rpl33*	207
*ndhE*	306	*psbF*	120	*rpl36*	114
*ndhJ*	477	*psbH*	222	*rps2*	747
*petA*	963	*psbI*	111	*rps4*	618
*petG*	114	*psbK*	186	*rps8*	435
*petL*	96	*psbM*	117	*rps11*	418
*psaA*	2,253	*psbN*	132	*rps18*	336

### Phylogenetic analyses of the Campanulidae

**Figure 3:**
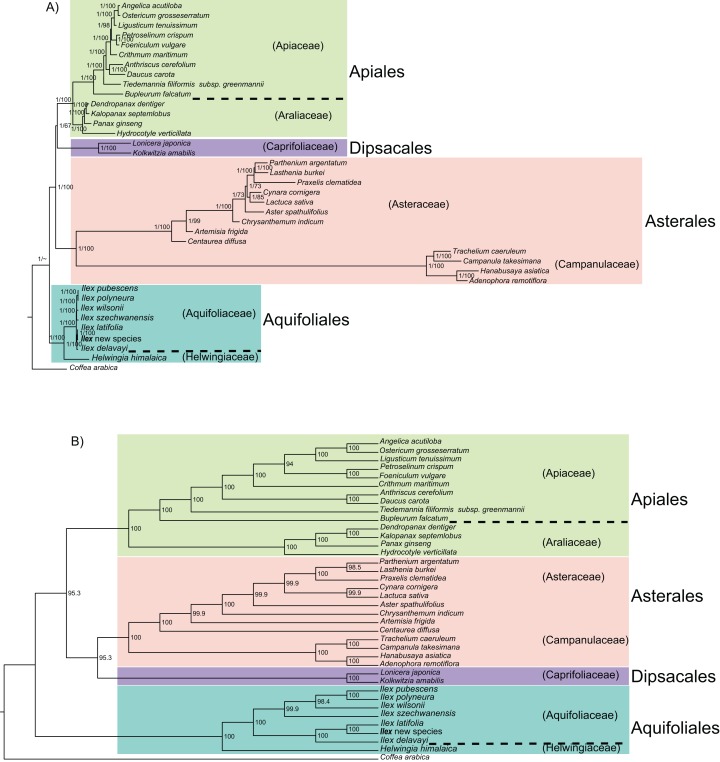
Phylogeny of 37 campanulid species using their complete chloroplast genomes. In subgraph (A) numbers near nodes (on left) indicate the Bayesian posterior probability and numbers near nodes (on right) indicate the maximum likelihood bootstrap values for each clade present in the 50% majority-rule consensus tree. In subgraph (B) numbers near nodes indicate the maximum parsimony bootstrap values for each clade present in the 50% majority-rule consensus tree.

The phylogeny produced from the analysis of 37 complete chloroplast genomes is well-supported, but while the results from ML and BI are congruent, the phylogeny from MP is not (Figs. 3A and 3B). With ML and BI, Aquifoliales are basal, Asterales are the next branch, and the Dipsacales are sister to the Apiales. The six families with multiple species are all well-supported (Fig. 3A). In the MP phylogeny, however, the Dipsacales are sister to the Asterales, and the Apiales are the next branch (Fig. 3B).

**Figure 4:**
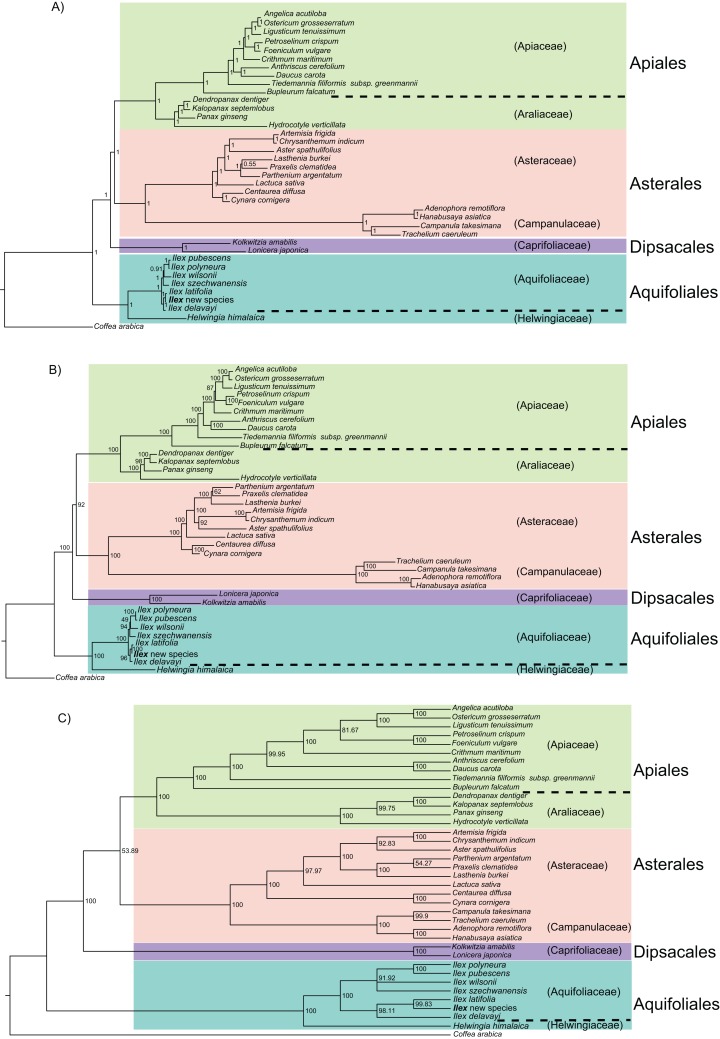
Phylogeny of 37 campanulid species using their 36 shared coding genes. In subgraph (A) numbers near nodes indicate the Bayesian posterior probability. In subgraph (B) numbers near nodes indicate the maximum likelihood bootstrap values for each clade present in the 50% majority-rule consensus tree. In subgraph (C) numbers near nodes indicate the maximum parsimony bootstrap values for each clade present in the 50% majority-rule consensus tree.

The phylogeny based on 36 shared protein-coding genes has a consistent family-level topology in analyses with BI, ML, and MP. The Aquifoliales are still basal, but the Dipsacales are the next branch, and the Asterales are sister to the Apiales (Fig. 4). However, within the Asteraceae, the topology from BI is different from those from ML and MP, and MP also did not resolve the relationships of *Ilex wilsonii* and *Ilex szechwanensis* (Fig. 4C).

## Discussion

The only other published chloroplast genomes in the order Aquifoliales are for seven species of *Ilex* in the family Aquifoliaceae (Yao et al., 2016). The length of the *Helwingia* genome (158,362 bp) is similar to that of *Ilex* (157,610–157,918 bp) (see Table 1 in Yao et al., 2016). *Helwingia* (94 genes) has two fewer protein coding genes than *Ilex* (96) while both have the same number of tRNA (40) and rRNA (eight) genes.

Minor differences among the published chloroplast genomes are common, including gene loss or gain events, but these do not separate *H. himalaica* from the others. Both *H. himalaica* and *Helianthus annuus* have *ycf15* (Timme, 2009), but *Guizotia abyssinica* does not (Dempewolf et al., 2010). *H. himalaica* and *L. sativa* have trnE (Kanamoto et al., 2004) but *H. annuus* and *G. abyssinica* do not. Gene *rps16* has one intron in *G. abyssinica*, *H. annuus*, and *L. sativa*, but none in *H. himalaica*, while gene *ycf3* has two introns in *H. himalaica*, *G. abyssinica*, and *H. annuus*, but none in *L. sativa*. Gene *ycf15* is between *ycf2* and *trnL-CAA* in *H. himalaica*, but between *rps7* and *trnV-GAC* in *H. annuus*. Gene *ndhF* is in the IRb-SSC junction in both *L. sativa* and *H. himalaica*, but in the SSC-IRa junction in *G. abyssinica* and *H. annuus*. The lengths of the published chloroplast genomes for the Campanulaceae range from 162,321 bp (*T. caeruleum*, Haberle et al., 2008) to 171,724 bp (*Adenophora remotiflora*, Kim et al., 2016) and are longer than those of other campanulid species. Kim et al. (2016) attribute this longer length to expansion occurring in the IR and LSC regions as well as the gene arrangements.

The many mononucleotide SSRs identified in *H. himalaica* are potentially useful for studies of the evolutionary history of populations (Khadivi-Khub et al., 2014; Chae et al., 2014). The dominance of A/T in mononucleotide SSRs in *Helwingia* is similar to other published studies (Huang et al., 2014; Kuang et al., 2011). It has been suggested that repeated sequences play an important role in genomic rearrangement and sequence variation in chloroplast genomes (Huang et al., 2014; Yang et al., 2013). Approximately 63% of repeats were found in intergenic regions which are often also divergent hotspot regions (e.g. Yao et al., 2015; Huang et al., 2014), showing the potential of these regions for the development of new phylogenetic markers for species identification in *Helwingia* and related genera in the Aquifoliales.

Massive rearrangements in the chloroplast genome have been identified in the Campanulaceae in comparison with other campanulid families (Fig. 2). Except for gene block 1 and 2, most gene blocks in the LSC have been rearranged, including changes in gene order and transcribing direction (Fig. 2). The chloroplast gene rearrangement in Campanulaceae was first identified in *T. caeruleum*, and inferred as the effects of recombination of repeats or tRNA genes (Haberle et al., 2008). Comparing with other angiosperm chloroplast genomes, more repeats and tRNA genes occurred near rearrangement endpoints in this species. The positive connection between rearrangement and repeated sequences has also been found in other plants, like *Arbutus unedo* (Martínez-Alberola et al., 2013), Geraniaceae (Weng et al., 2013), *Vaccinium macrocarpon* (Fajardo et al., 2013) and cupressophytes (Wu & Chaw, 2014). However, the effects of these chloroplast gene rearrangements on plant physical functions still need more study.

The phylogenetic trees based on complete chloroplast genomes are incongruent with those from the protein-coding genes. Aquifoliales are basal in all phylogenetic analyses, but the phylogenetic relationships among the Asterales, Apiales and Dipsacales differ in different analyses (Figs. 3 and 4). The phylogeny based on complete chloroplast genomes using BI and ML methods found that the Apiales are sister to the Dipsacales (Fig. 3A), which agrees with recent phylogenies for this subclass based on other markers (Beaulieu, Tank & Donoghue, 2013; Wikström et al., 2015; Chen et al., 2016; The Angiosperm Phylogeny Group, 2016). However, using the MP method with the same data resulted in a phylogeny with the Asterales sister to the Dipsacales (Fig. 3B). The phylogenies based on protein-coding genes found that the Apiales are sister to Asterales with all three methods, although the topology within the Asteraceae differed between BI and the other two methods (Fig. 4). Three orders (of seven) and 22 families (of 29) in the subclass Campanulidae could not be included in our analyses because there are no published complete chloroplast genomes for these clades, which emphasizes the need for increased coverage of angiosperm orders and families in future studies of chloroplast genomes.

## Conclusion

We report the chloroplast genome of *H. himalaica* as the first in the Helwingiaceae and the second genus in the Aquifoliales. It has the typical quadripartite circular structure, including an LSC with 87,810 bp and an SSC with 18,560 bp, separated by a pair of IR regions with 25,996 bp. In total, 142 genes were detected in this genome, consisting of 94 protein-coding genes, 40 tRNA, and eight rRNA. Repeated sequences are mainly distributed in intergenic regions. Comparisons among the available chloroplast genomes within the campanulids reveal massive chloroplast gene rearrangement in the Campanulaceae. The phylogenetic relationships among Apiales, Asterales and Dipsacales were incongruent between phylogenetic results produced from complete chloroplast genomes and the 36 shared protein-coding genes. The topology within Asteraceae also varied, which shows that further studies are still needed in these three orders. The results of this study will facilitate understanding of not only the family Helwingiaceae and its relationships with other taxa in the Aquifoliales, but also phylogenetic relationships within the angiosperms at higher levels.

## Supplemental Information

10.7717/peerj.2734/supp-1 Supplemental Information 1 Numbers of different kinds of SSR detected in the chloroplast genome of *Helwingia himalaica*.

10.7717/peerj.2734/supp-2 Supplemental Information 2 Primers validating four junction regions in resulting chloroplast genome of *Helwingia himalaica*.

## Additional Information and Declarations

### DNA Deposition

The following information was supplied regarding the deposition of DNA sequences: GenBank KX434807.
