# Supplementary material for: The complete chloroplast genome sequence of Helwingia himalaica (Helwingiaceae, Aquifoliales) and a chloroplast phylogenomic analysis of the Campanulidae
Source: PeerJ. 2016 Nov 29;4:e2734. doi: 10.7717/peerj.2734 (PMC5131622; doi:10.7717/peerj.2734)

**Supplementary Fig. 1** Numbers of different kinds of SSR detected in the chloroplast genome of *Helwingia himalaica*.


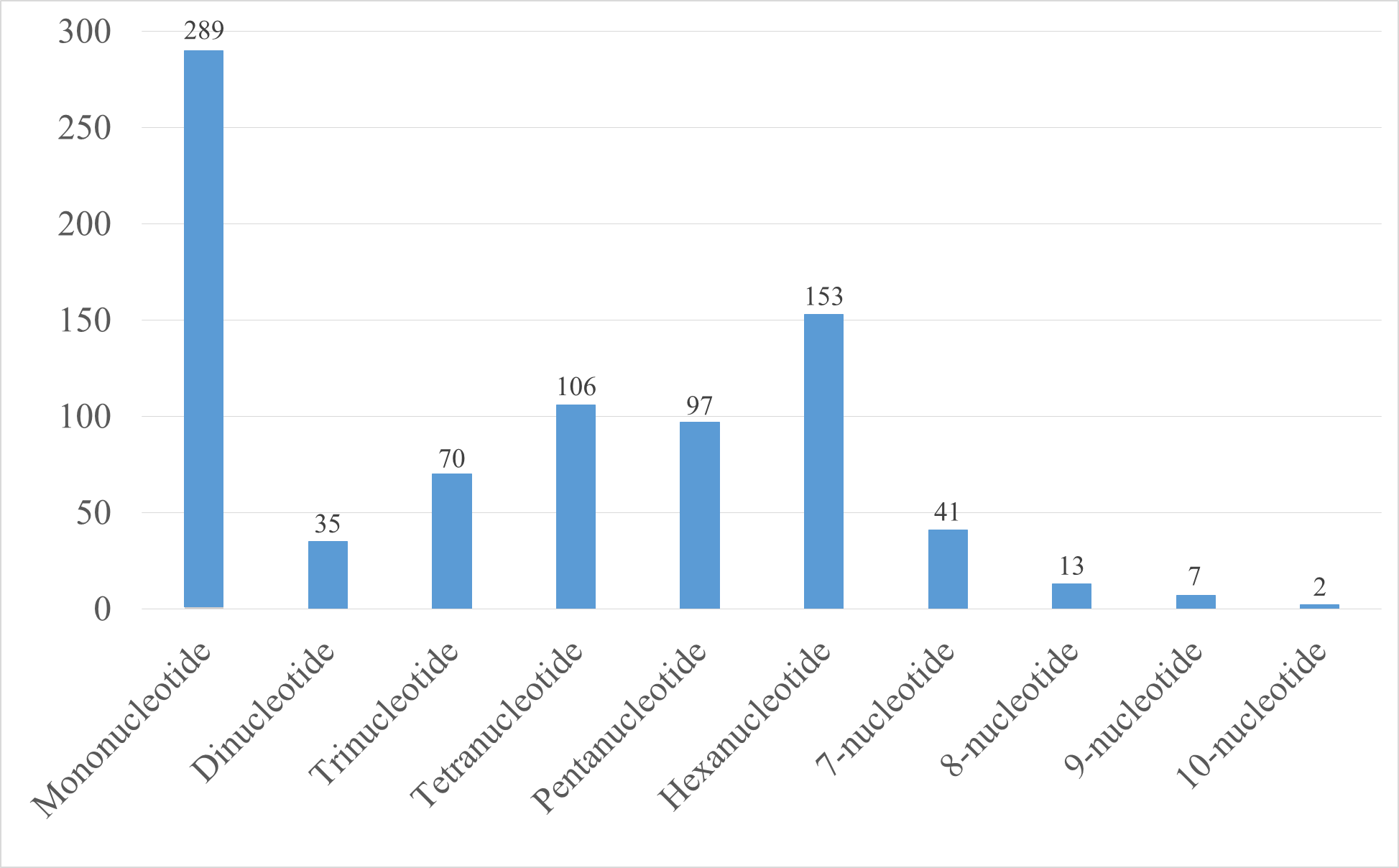

Supplement: Supplemental Information 1 [file peerj-04-2734-s001.docx]
