# Supplementary material for: The complete chloroplast genome sequence of Helwingia himalaica (Helwingiaceae, Aquifoliales) and a chloroplast phylogenomic analysis of the Campanulidae
Source: PeerJ. 2016 Nov 29;4:e2734. doi: 10.7717/peerj.2734 (PMC5131622; doi:10.7717/peerj.2734)
Supplement: Supplemental Information 2 [file peerj-04-2734-s002.docx]

**Supplementary Table 1.** Primers validating four junction regions in resulting chloroplast genome of *Helwingia himalaica*.

|  | **f** | **r** |
| --- | --- | --- |
| IRa-LSC | AGAGCCGGATCTAAGCGTTG | ACCTTGGTCTTAGTGTAGACG |
| LSC-IRb | TGGCCTACCATCCGATCTGT | GTCGGACAAGTGGGGAATGT |
| IRb-SSC | GGGGGAAAGTGCGGAAGAAA | GGGGGATAAGGGTACTGGCT |
| SSC-IRa | CGAGTTTTGCTAGGGTGAGC | AGCTTCTGAAACGAAGGGGA |
